# Platform construction and extraction mechanism study of magnetic mixed hemimicelles solid-phase extraction

**DOI:** 10.1038/srep38106

**Published:** 2016-12-07

**Authors:** Deli Xiao, Chan Zhang, Jia He, Rong Zeng, Rong Chen, Hua He

**Affiliations:** 1 Department of Analytical Chemistry, China Pharmaceutical University, Nanjing 210009, China; 2 Key Laboratory of Drug Quality Control and Pharmacovigilance, Ministry of Education, China Pharmaceutical University, Nanjing 210009, China

## Abstract

Simple, accurate and high-throughput pretreatment method would facilitate large-scale studies of trace analysis in complex samples. Magnetic mixed hemimicelles solid-phase extraction has the power to become a key pretreatment method in biological, environmental and clinical research. However, lacking of experimental predictability and unsharpness of extraction mechanism limit the development of this promising method. Herein, this work tries to establish theoretical-based experimental designs for extraction of trace analytes from complex samples using magnetic mixed hemimicelles solid-phase extraction. We selected three categories and six sub-types of compounds for systematic comparative study of extraction mechanism, and comprehensively illustrated the roles of different force (hydrophobic interaction, π-π stacking interactions, hydrogen-bonding interaction, electrostatic interaction) for the first time. What’s more, the application guidelines for supporting materials, surfactants and sample matrix were also summarized. The extraction mechanism and platform established in the study render its future promising for foreseeable and efficient pretreatment under theoretical based experimental design for trace analytes from environmental, biological and clinical samples.

In recent years, a new solid-phase extraction (SPE) method based on mixed hemimicelles assemblies (hemimicelles/admicelles) have been attracting more and more attention^1^,^2^,^3^,^4^,^5^. The employed sorbents are produced by adsorbing ionic liquid (such as 1- hexadecyl-3-methylimidazolium tetrafluoroborate) or ionic surfactants (such as sodium dodecyl sulfate and cetyltrimethylammonium bromide) on the surface of metal oxides (such as alumina, silica, titanium dioxide, and ferric oxyhydroxides). Compared with the normal sorbents, one feature of these sorbents is that the outer surface of hemimicelles is hydrophobic and the admicelles is ionic, which offers regions of distinct polarity, allowing the adsolubilization of diverse analytes. The inoic liquid or surfactant hydrocarbon chains provide hydrophobic or π-π stacking interactions for hydrophobic analytes, while the polar groups adsorb ionic analytes via electrostatic interaction or hydrogen-bonding interaction.

What makes hemimicelle-based SPE special is its versatility (types of interactions can be changed according to the region of the isotherm), the high number of sites for solubilization, and the dynamic character of the surfactant or ionic liquid aggregates adsorbed onto the mineral oxide. Any scientist with a good basis on the chemistry involved can select the best supporting materials and surfactant for a specific application. Up to now, Various SPE methods based on mixed hemimicelles have been described in the literatures. Early in 2003, Merino^6^ *et al*. firstly developed a simple and reliable SPE method for the identification and quantitation of benzalkonium surfactants based on their adsorption on sodium dodecylsulphate (SDS)-alumina mixed hemimicelles assemblies in sewage and river water complex matrixes, demonstrating that this approach is an interesting alternative for the concentration of amphiphiles in complex matrixes. In 2006, Cantero^7^ *et al*. successfully used hemimicelles/admicelles of SDS-alumina and cetylpyridinium chloride (CPC)-silica as sorbent materials and filled it in the SPE cartridge columns for the concentration of alkylphenol polyethoxylate (APE) biodegradation products from wastewater and river water samples. Niu^3^ *et al*. adopted the cationic surfactant cetyltrimethylammonium bromide (CTAB)-titanate nanotube system to form hemimicelles/admicelles for extracting nonpolar phthalate esters. CTAB-titanate nanotubes admicelles and CTAB-titania admicelles were compared, since possessing higher surface area and more ion exchangeable OH^−^ groups in the interlayer and surface, titanate nanotubes materials were demonstrated to be solid-phase extraction adsorbents to pre-treat water samples.

Recently, room temperature ionic liquids (RTILs) have attracted great attentions due to their unique chemical and physical properties. Li^8^ *et al*. firstly attempted to prepare mixed hemimicelles by adsorbing 1-hexyl-3-methylimidazolium bromide ([C_6_mim]Br) and 1-dodecyl-3-methylimidazolium bromide ([C_12_mim]Br) on silica surface for the preconcentration of five phthalates in environmental water sample. Good recovery and precision were obtained, which indicated that RTILs could be applied in the mixed hemimicelles SPE method. Generally speaking, inoic liquid or surfactant-coated mineral oxides columns for SPE have many advantages, such as high extraction efficiency, environmentally benign, excellent solvation qualities, high thermal stability and so on, making them become a promising tool for the preconcentration of organic compounds in the complex matrix. However, traditional SPE technique based on mixed hemimicelles requires column packing, resulting in cumbersome steps and time-consuming operation. Moreover, the most widely used support materials for hemimicelles and admicelles such as alumina, silica, titanium dioxide usually have large particles and relatively small surface area, which limits the large-scale application of mixed hemimicelles solid phase extraction techniques.

To overcome the above problem, a new type of sample enrichment technique on the basis of magnetic mixed hemimicelles SPE has been proposed^9^,^10^,^11^,^12^,^13^. Magnetic nanomaterials were adopted as the support materials and combined with RTILs to form the mixed hemimicelles. Magnetic nanomaterials possess a high surface area and strong magnetism, and they can be assumed that their use in analytical chemistry can improve the adsorption capacity of analytes and avoid the time-consuming enrichment process of loading large volume samples. Owing to reusable adsorbents and seldom-used organic solvents, magnetic mixed hemimicelles SPE has been widely used in the rapid analysis of complex trace substances in aqueous systems. In recent years, the magnetic mixed hemimicelles SPE has developed dramatically and has been reported widely^13^,^14^,^15^,^16^,^17^,^18^. Sun^15^ *et al*. firstly introduced magnetic nanomaterials into the mixed hemimicelles to form mixed hemimicelles sorbents in which positively charged CTAB ions were adsorbed onto negatively charged magnetic Fe_3_O_4_ NPs in weak basic media for the preconcentration of four sulfonamides compounds in five environmental water samples. Zhu^16^ *et al*. developed the Fe_3_O_4_/SiO_2_ core/shell nanoparticles and modified them by coating with cationic surfactants (CTAB) to form the nanosized SPE sorbents which combined the advantages of mixed hemimicelles and silica-coated magnetic nanoparticles for extraction and preconcentration of the active compounds in biological samples. Cheng^17^ *et al*. firstly introduced magnetic carbon nanotubes into mixed hemimicelles SPE to establish a novel mixed hemimicelles solid phase extraction (SPE) based on magnetic carbon nanotubes (MCNTs) and ionic liquid (IL) for fast extraction of trace natural substances from bio-matrix samples. In this novel SPE, the formation of C_16_mimBr with mixed hemimicelles on the surface of MCNTs@SiO_2_ nanoparticles (NPs) causes retention of analytes by strong hydrophobic, π-π stacking interactions and electrostatic interactions.

Although recent studies have reported magnetic mixed hemimicelles technique for extraction and preconcentration of trace compounds in complex aqueous solutions^12^,^18^, their objectives were how to establish a magnetic mixed hemimicelles SPE system sticking to just only one kind of compound, without making exploration, comparison and outlook systematically. Furthermore, there is no completely clear theory pertaining to magnetic mixed hemimicelles SPE mechanism hitherto. Even if there were sporadic speculation, systematic experiment datum are still insufficient. So up to now, the research of magnetic mixed hemimicelles SPE is still on the stage of taking a part for the whole, lacking of solid theoretical guidance and provident experimental design. In sight of this, we groped pros and cons of magnetic mixed hemimicelles SPE technique when applied on trace compounds extraction and detection in complex aqueous systems, established magnetic mixed hemimicelles SPE platform and made further research on the extraction mechanism. We selected three categories and six sub-types of compounds according to the mixed hemimicelles solid phase extraction mechanism (hydrophobic interaction, electrostatic interaction, hydrogen-bonding interaction and π-π stacking interactions, etc.). They are hydrophobic compounds containing aromatic ring (flavonoids and chlorophenol compounds), hydrophilic compounds containing aromatic ring (gatifloxacin and cephalosporin compounds) and hydrophobic compounds excluding aromatic ring (retinyl acetate and myristic acid). Firstly, these different kinds of compounds were investigated and validated to make sure that if they were suitable for the magnetic mixed hemimicelles SPE system. Then the extraction mechanism of the magnetic mixed hemimicelles SPE system was studied systematicly by comparing the extraction recovery and structure of the compounds, which provides guidelines for subsequent researchers. Meanwhile, a series of ionic liquids including different carbon chain lengths, different nucleus and different anions were selected and investigated on the extraction efficiency of the flavonoids, which also provide evidences for the extraction mechanism of the magnetic mixed hemimicelles SPE system. At last, the magnetic mixed hemimicelles SPE system was applied for the extraction and preconcentration of the different trace compounds in complex aqueous systems. A universal extraction platform is promising to be constructed based on the extraction mechanism study.

## Method

### Chemicals and materials

All reagents were of analytical reagent grade. Five flavonoids (luteolin, quercetin, kaempferol, apigenin and chrysin) and three chlorophenols (4-chlorophenol, 2,4-dichlorophenol and 2,4,6-trichlorophenol) standards were purchased from Sinopharm Chemical Reagent Co., Ltd, China. Their chemical structures are as shown in Table 1. Three fluoroquinolones (ofloxacin, ciprofloxacin, gatifloxacin) and three cephalosporins (ceftazidime, cefotaxime, cephradine) standards were purchased from Sinopharm Chemical Reagent Co, Ltd, China. Their chemical structures are as shown in Table 2. Retinol acetate and myristic acid standards were purchased in China Sinopharm Chemical Reagent Co, Ltd., and their chemical structures are as shown in Table 3. Cetyl trimethyl ammonium bromide (CTAB) was purchased from Beijing Chemical Reagent Company. 1-hexadecyl-3-methylimidazolium bromide (C_16_mimBr), 1- hexadecyl-3-methylimidazolium tetrafluoroborate (C_16_mimBF_4_), 1-dodecyl-3- imidazole tetrafluoroborate (C_12_mimBF_4_), 1-octyl-3-methylimidazolium tetrafluoroborate (C_8_mimBF_4_), 1-butyl-3-methylimidazolium tetrafluoroborate (C_4_mimBF_4_)), and N-cetylpyridinium bromide were purchased from CAS Lanzhou Institute of Chemical Physics, and their chemical structures are as shown in Table 4. Ferric chloride hexahydrate (FeCl_3_.6H_2_O), ethylene glycol (EG), diethylene glycol (DEG), sodium acrylate (CH_2_=CHCOONa), sodium acetate (CH_3_COONa), disodium hydrogen phosphate (NaH_2_PO_4_) and phosphoric acid sodium dihydrogen phosphate (Na_2_HPO_4_) were purchased from Aladdin.

### Instruments

The morphology of the as-synthesized nanoparticles was studied by using a FEI TECNAI G2F20 transmission electron microscope (TEM). The surface groups on the as-synthesized nanoparticles were measured with a 8400 s FT-IR spectrometer (Shimadzu Corporation, Japan) by using KBr as diluting agent. The magnetic properties were studied by using a LDJ 9600-1 vibrating sample magnetometer (VSM) operating at room temperature with applied fields up to 10 kOe. Zeta-potential measurements of as-synthesized nanoparticles were performed with ZetaPlus Zeta Potential Analyzer (Brookhaven, USA).

### Preparation of magnetic Fe_3_O_4_ NPs

Fe_3_O_4_ NPs were prepared by solvothermal method^19^. Briefly, 2.4 g FeCl_3_·6H_2_O, 3.4 g CH_2_CHCOONa and 3.4 g CH_3_COONa were added into a mixture of ethylene glycol (EG, 22.5 mL) and diethylene glycol (DEG, 22.5 mL) under ultrasonication for about 1 h. The resulting homogeneous black solution was then transferred and sealed into a teflon-lined stainless-steel autoclave. The autoclave was heated at 200 °C for 10 h, and then cooled to room temperature. After the reaction, the obtained Fe_3_O_4_ NPs were washed with ethanol and water for several times, and then dried in vacuum at 60 °C for 10 h.

### Magnetic solid phase extraction (MSPE) based on mixed hemimicelles

**Figure 1:**
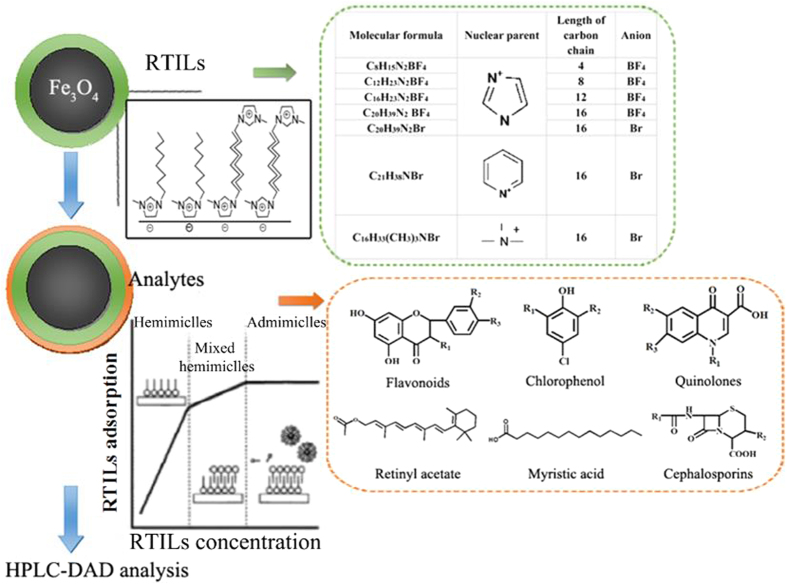
Scheme of the preparation of surfactant-coated Fe_3_O_4_ NPs and its application as SPE sorbents for extraction and preconcentration of analytes in aqueous system.

As shown in Fig. 1, the MSPE procedure based on mixed hemimicelles using Fe_3_O_4_ NPs allowed rapid preconcentration of analytes in complex aqueous systems by a simple magnetic extraction method. First, a certain amount of Fe_3_O_4_ NPs, C_16_mimBr and 2 mL of phosphate buffer were added sequentially into a 4 mL centrifuge tube. The mixture was sonicated for 5 min to suspend the Fe_3_O_4_ NPs to form mixed hemimicelles, then 2 mL aqueous sample was added in the mixed solution. In order to absorb analytes, the mixture was sonicated for 5 min, and then equilibrated for 90 min in an oscillator. In the external magnetic field, the magnetic nanomaterial with analytes was adsorbed onto the centrifuge tube wall. When the suspension became limpid (about 2 s later), the supernatant was decanted, and the isolated nanoparticles were eluted with 1 mL methanol containing 1% acetic acid (HAc) (0.5 mL every time and washed twice) to desorb the preconcentrated target analytes. The eluted solution was collected and then dried under a stream of nitrogen at 60 °C, and redissolved in 500 μL methanol. The methanol solution was vibrated in an ultrasonic bath for 2 min in order to insure that the target analytes can be dissolved into methanol completely. Finally, after filtration through 0.45 μL membrane, 10 μL of the solution was injected into the HPLC system for analysis.

For the contrast study of methods, we extracted flavonoid compounds by magnetic retrieval of chitosan solid-phase extraction technique at the same time. Firstly, 1 mL sample was added into a 5 mL vessel, the pH of which was adjusted to 7.0 by using 0.02 M PBS buffered solution. Secondly, 15 mg chitosan was added into the above solution and was oscillated slightly for 30 s. And then a certain amount of Fe_3_O_4_ NPs was added, the mixture was sonicated for 1 min. In the external magnetic field, the chitosan Fe_3_O_4_ composites adsorbed with analytes was isolated from solution. After 2 s the suspension became limpid, the supernatant was decanted, and the collected nanoparticles were eluted with 1 mL acetonitrile containing 1% HAc to desorb composite. Finally, the eluted solution was collected and then dried under a stream of nitrogen at 60 °C, and redissolved in 500 μL methanol. The methanol solution was vibrated in an ultrasonic bath for 2 min in order to insure that the target analytes can be dissolved into methanol completely. After filtration through 0.45 μL membrane, 10 μL of the solution was injected into the HPLC system for analysis.

### Chromatographic conditions

Five flavonoids (luteolin, kaempferol, quercetin, apigenin and chrysin) were separated and quantified by a high performance liquid chromatography with an automatic sampler (Agilent-1260). The analytical column was a ZORBAX Eclipse XDB-C_18_ column (4.6 mm × 150 mm, 5 μm) supplied by Agilent. The gradient elution was as follows: mobile phase A was methanol, mobile phase B was 0.2% aqueous phosphoric acid. The ratio of A was 48% at the beginning, linear gradient from 48–45% A in 8–12 min, keep 45% A in 12–18 min, linear gradient from 45–60% A in 18–22 min, linear gradient from 60–80% A in 22–30 min. Other general chromatographic conditions were shown in Table 5 (similarly hereinafter).

Three chlorophenols (4-chlorophenol, 2,4-dichlorophenol and 2,4,6-trichlorophenol) were quantified by HPLC-DAD analysis and the mobile phase was methanol-0.1% formic acid (25: 75, V/V). Three fluoroquinolones (ofloxacin, ciprofloxacin, gatifloxacin) were quantified by high-performance liquid chromatography with diode array detector (HPLC-DAD) and the mobile phase was methanol - 1% aqueous solution of triethylamine (adjusted with phosphoric acid to pH 4.3) (25: 75, V/V). Three cephalosporins (ceftazidime, cefotaxime, cephradine) were quantified by HPLC-DAD analysis and the mobile phase was methanol-0.1% formic acid (25:75, V/V). Vitamin A acetate was quantified by HPLC-DAD analysis and the mobile phase was methanol - water (96: 4, V/V). Myristic acid was quantified by HPLC-RID quantitative analysis. HPLC system (Shimadzu LC-20AT), comprising a quaternary pump, column oven, autosampler and quaternary detectors, in which columns produced by Agilent ZORBAX Eclipse XDB-C_18_ column (150 mm × 4.6 mm, 5 μm). The mobile phase was methanol - water (90:10, V/V).

## Results and Discussion

### Characterization

**Figure 2:**
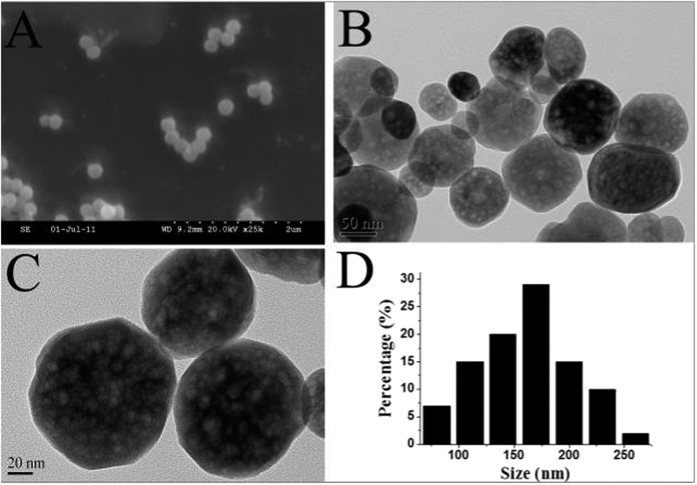
SEM (**A**) and TEM (**B**,**C**) image of Fe_3_O_4_ NPs, the corresponding particle size distribution histogram (**D**).

The TEM image and SEM image of the morphology of the Fe_3_O_4_ NPs were shown in Fig. 2. It can be seen that most of the particles were uniform with a spherical shape from different angles as reported before. As shown in Fig. 2(A), the TEM image suggested that the size of the particle was about 170 nm in diameter. In the present experiment, it can be uniformly suspended in solution during the extraction process, and therefore suitable for magnetic mixed hemimicelles solid phase extraction.

**Figure 3:**
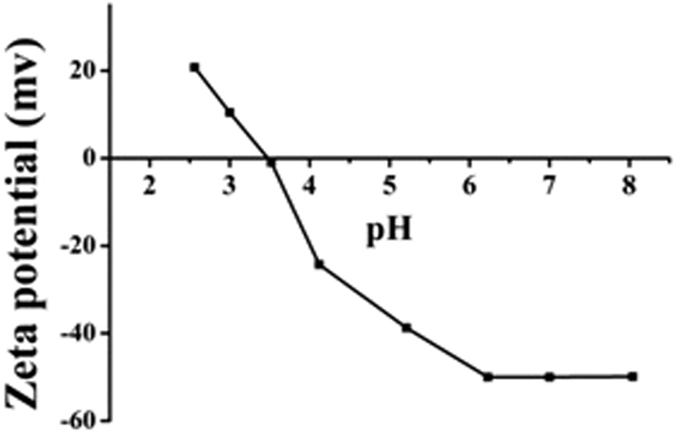
Zeta-potential at different pH of Fe_3_O_4_ NPs.

For the metal oxide, the isoelectric point measured by zeta-potential is an important property^20^. The study shows that the charge density and the type of metal oxide will change with the pH of the buffer solution. When the pH is below the isoelectric point, Fe_3_O_4_ NPs surface is positively charged, otherwise negatively charged. As shown in Fig. 3, the isoelectric point (IEP) of Fe_3_O_4_ NPs was found to be at pH 3.47, which approached to previously reported data for natural silica (3.0)^12^. When the pH is 6, the Fe_3_O_4_ which is negatively charged produced the electrostatic adsorption with ionic liquid to form the magnetic mixed hemimicelles SPE system.

**Figure 4:**
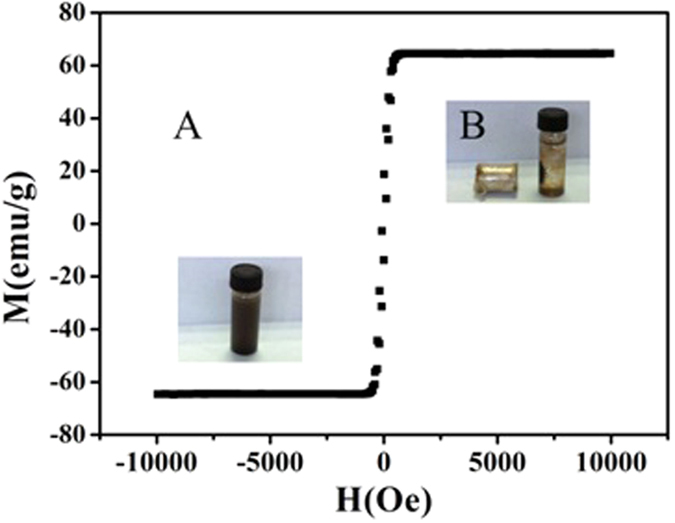
VSM magnetization curves of Fe_3_O_4_ NPs at room temperature (**A**) and the magnetic performance of Fe_3_O_4_ NPs within 30 seconds using an external magnet (**B**).

Figure 4 showed VSM magnetization curves of Fe_3_O_4_ NPs at room temperature. The maximum saturation magnetization was 64.60 emu g^−1^ for Fe_3_O_4_ NPs which indicated their magnetism was sufficient for MSPE. Figure 4 also demonstrated that the prepared Fe_3_O_4_ NPs could be separated from their dispersion quickly once an external magnetic field was applied.

**Figure 5:**
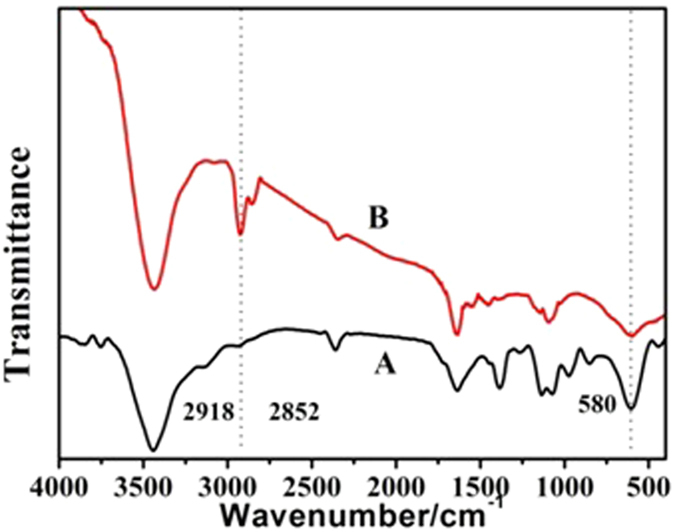
FT-IR spectra of Fe_3_O_4_ NPs (**A**) and C_16_mimBr-coated Fe_3_O_4_ NPs (**B**).

For demonstrating C_16_mimBr was coated on the surface of Fe_3_O_4_ NPs successfully, Fe_3_O_4_ NPs and C_16_mimBr-Fe_3_O_4_ NPs were characterized by Fourier transform infrared spectrometer. As shown in Fig. 5, both (A) and (B) displayed many common characteristics in their spectra. Such as the typical peaks around 3421 cm^−1^, which were ascribed to the stretching vibration of O-H band. However, the stretching vibration of C-H band at 2918 and 2852 cm^−1^ and the vibration of Fe-O at 583 cm^−1^ appeared in the spectrum of the C_16_mimBr-coated Fe_3_O_4_ NPs, as shown in Fig. 5(B), implying that C_16_mimBr was successfully adsorbed onto the surface of Fe_3_O_4_ NPs.

### Zeta-potential isotherm

**Figure 6:**
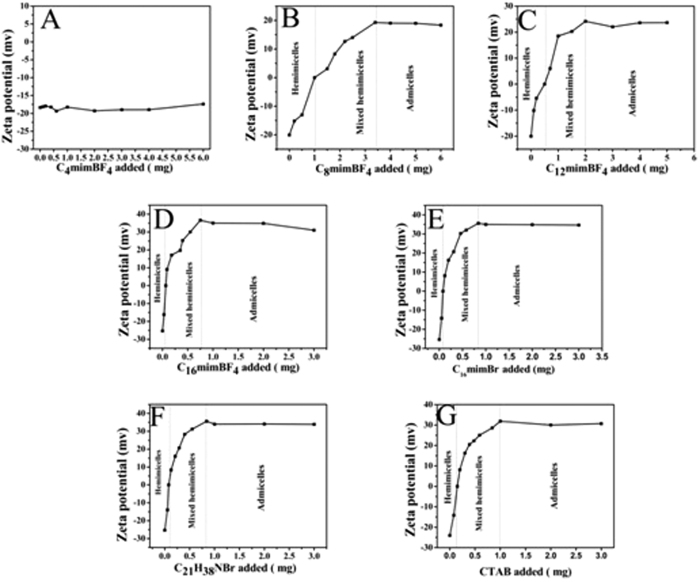
Zeta-potential of Fe_3_O_4_ NPs by adsorption of (**A**) C_4_mim BF_4,_ (**B**) C_8_mimBF_4_, (**C**) C_12_mimBF_4_, (**D**) C_16_mimBF_4_, (**E**) C_16_mimBr, (**F**) C_21_H_38_NBr and (**G**) CTAB.

The adsorption isotherms of C_16_mimBr on Fe_3_O_4_ NPs are useful for not only understanding the principle of mixed hemimicelles but also selecting the optimal surfactant amount, which can be demonstrated by zeta-potential isotherm^8^,^21^. Figure 6 presents the zeta-potential change of Fe_3_O_4_ NPs suspensions when C_16_mimBr is absorbed at pH 6.0. Generally, the isotherms can be divided into three regions including hemimicelles, mixed hemimicelles and admicelles. And the process involves four adsorption mechanisms such as hydrophobic interaction, electrostatic interaction, hydrogen-bonding interaction and π-π stacking interactions. As shown in Fig. 6(D), in the first region (C_16_mimBr added from 0 to 0.1 mg), due to electrostatic attraction, single-layer coverage (hemimicelles) is gradually formed, while the added ionic surfactant molecules are adsorbed onto the surface of negatively charged Fe_3_O_4_ NPs under pH 6.0 (above the IEP). The zeta-potential of mineral oxides increases from negative to zero. In the second region, the formation of bilayers admicelles involves hydrophobic and electrostatic interactions. With the increasing amount of ionic surfactant molecules, the zeta-potential of mineral oxides reverses and increases after passing through a zero zeta-potential. Both the above-mentioned regions are suitable for SPE method. When the amount of C_16_mimBr is above 0.8 mg, the zeta-potential of mineral oxides remains constant, manifesting that the entire surface of metal oxides is saturated and surfactants begin to form micelles in solution. In this region, aqueous surfactant micelles could be formed in the aqueous solution and be in equilibrium with admicelles, which may result in the solubilization of analytes into micelles in bulk solution again and cause the isotherm region span unsuitable for SPE. However, it should be pointed out that the structure of the surfactant aggregates could not be simply defined as monolayers (hemimicelles) or bilayers (admicelles), which need further evidences for the formation of discrete surface aggregates.

As shown in Fig. 6(B,C,D,F and G), the trend of the formation of the mixed hemimicelles is basically similar, the longer the hydrophobic chain is, the less ionic liquid is needed. From the Fig. 6(A), it can be seen that the zeta potential of Fe_3_O_4_ NPs cannot be affected by the amount of the C_4_mimBF_4_, which might be explained by the fact that the C_4_mimBF_4_ has poor affinity with Fe_3_O_4_ NPs and are more difficult to form micelles because of short carbon chain. So they are not appropriate for magnetic mixed hemimicelles solid phase extraction.

### Study of extraction mechanism

In recent years, regarding to the extraction mechanism of the magnetic mixed hemimicelles SPE, there are sporadic speculation including hydrophobic interaction, electrostatic interaction, hydrogen-bonding interaction and π-π stacking interactions. In order to fully elaborate extraction mechanism, we carried out a systematic investigation by using the following three categories and six sub-types of compounds.

### Research on hydrophobic compounds containing aromatic ring (flavonoids and chlorophenol compounds)

In this work, two sub-types of compounds consisting of five flavonoids and there chlorophenol were examined. As shown in Table 1, these two sub-types of compounds both have high hydrophobicity constant (log P), and also have a gap between five flavonoids and three chlorophenol compounds in the hydrophobicity constant respectively. So the hydrophobic interaction can be investigated in the magnetic mixed hemimicelles solid phase extraction. More importantly, though flavonoids and chlorophenol compounds all have relatively high hydrophobicity constant, there are some differences in their structures. Compared to the chlorophenol compounds, the flavonoids posses a longer aromatic chain, by which the π-π stacking interactions on the magnetic mixed hemimicelles solid phase extraction can be obtained. In the study, the C_16_mimBr coated on the surface of the magnetic Fe_3_O_4_ NPs was chosen as solid phase adsorbents, and five flavonoids and three chlorophenol compounds were studied and compared.

**Figure 7:**
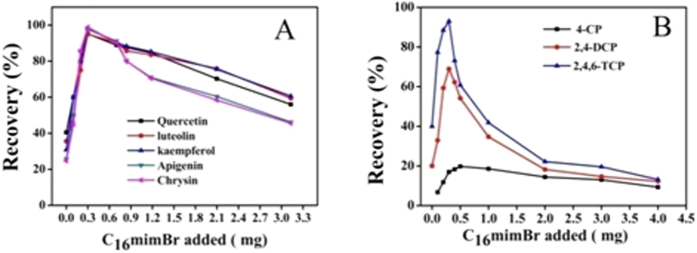
Effect of the amount of C_16_mimBr on the adsorption of flavonoids compounds (**A**) and chlorophenol compounds (**B**).

As shown in Fig. 7(A), in the absence of C_16_mimBr, the flavonoids were hardly adsorbed onto the surface of Fe_3_O_4_ NPs, because they only exist weaker hydrogen-bonding interaction and electrostatic repulsion at present. In contrast, with the increasing amount of C_16_mimBr, the adsorption ratio of five flavonoids increased remarkably. In the first region (0–0.1 mg of C_16_minBr, hemimicelles) the sorbent offer four types of interactions: hydrophobic interactions from the hydrocarbon chain, π-π stacking interactions from aromatic ring, hydrogen-bonding interaction and electrostatic repulsion from the uncovered groups in the mineral oxide surface. In the second region (below CMC, mixed hemimicelles) the sorbent offer three types of interactions: hydrophobic interactions from the hydrocarbon chain, π-π stacking interactions from aromatic ring and electrostatic attraction between C_16_minBr and flavonoids. In the third region (above CMC, admicelles), the recovery of flavonoids decreased due to the formation of micelles in the bulk aqueous solution, which led to a redistribution of the flavonoids into the solution. It is worth noting that when the concentration of C_16_mimBr is above CMC, the recovery of the apigenin and chrysin is relatively lower than the other three flavonoids compounds. It can be explained that the more hydrophobic apigenin and chrysin is, the more prone to interact with the redundant C_16_mimBr which is in the aqueous solution.

For three chlorophenol compounds, the trend of the recoveries is similar to the flavonoids. The recoveries increased with increasing C_16_mimBr, thus reaching a maximum level when 0.4 mg C_16_mimBr coats on the surface of the 8 mg Fe_3_O_4_ NPs. However, the recovery of 4-chlorophenol always maintained unsatisfactory even when increasing the amount of the C_16_mimBr. Under optimum conditions, the maximum adsorption rate of 2,4,6-trichlorophenol, 2,4-dichloro phenol, 4-chlorophenol can reach to 90%, 70% and 20%, respectively. Through literature review and analysis^14^, we found that Log P of 2,4,6-trichlorophenol, 2,4-dichlorophenol, 4-chlorophenol were 3.48, 2.88, 2.27 respectively. Therefore, it was speculated that with the amount of chlorine atom decreasing, the log P of chlorophenol compounds decreased and the hydrophobic properties of the chlorophenol compounds decreased gradually, thus the effect between compounds and mixed hemimicelles weakened by degrees, leading to the result that the recovery of 4-chlorophenol was below than that of 2,4,6-trichlorophenol, 2,4-dichlorophenol.

When conducting the overall comparison between the two sub-types of the compounds, it can be seen that the recovery of flavonoids is generally higher than the chlorophenol compounds. Particularly, it is unusual that 4-chlorophenol hydrophobic constant (2.27) is higher than that of quercetin (2.16), while the extraction recovery (20%) was significantly lower than that of quercetin (90%), suggesting that the hydrophobic constant cannot fully determine the level of extraction efficiency. The hydrophobic constant (Log P) value refers to partition coefficient values in n-octanol/water two-phase system, which only considers partition coefficient in two phases, without considering the characteristics of the compound structure. By comparing their structures, the flavonoids have larger aromatic system than chlorophenol compounds, making it easier to produce π-π stacking interactions with aromatic structure of ionic liquids (such as imidazole ring). These results suggest that it is necessary to consider the impact of hydrophobic interactions and π-π stacking interactions when investigating the mechanism of the magnetic mixed hemimicelles solid phase extraction.

### Research on hydrophilic compounds containing aromatic ring (quinolone and cephalosporin compounds)

**Figure 8:**
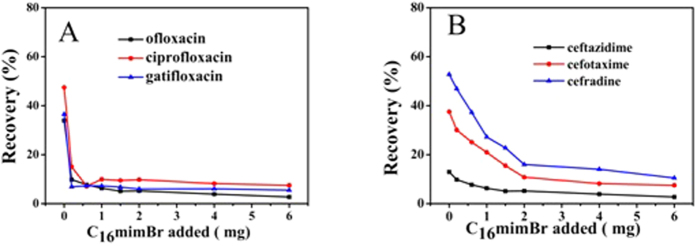
Effect of the amount of C_16_mimBr on the adsorption of Fluoroquinolone compounds (**A**) and Cephalosporin compounds (**B**).

To examine the impact of electrostatic interactions further, two sub-types of hydrophilic compounds (including three quinolone compounds and three cephalosporins compounds) were selected. As shown in Table 2, the hydrophobic constants (log P) of the two sub-types of compounds are relatively low. The effect of the C_16_mimBr was studied by varying the amount of the C_16_mimBr from 0 to 6 mg, which was shown in the Fig. 8. In the absence of C_16_mimBr, pure Fe_3_O_4_ NPs possess a certain adsorption towards the quinolone and cephalosporin compounds, because they exist weaker electrostatic attraction and hydrogen-bonding interaction at present. However, with the increasing amount of ionic liquids, the recoveries of the two kinds of compounds decreases, which may be attributed to the fact that the C_16_mimBr changed the charge properties of sorbents surface. Therefore the interactions between sorbents and analytes changed from electrostatic attraction to electrostatic repulsion, resulting in the decreases of recoveries. This result demonstrated that quinolone and cephalosporin compounds are not suitable for the magnetic mixed hemimicelles solid phase extraction. It may reveal a very useful rule that trying to analyze hydrophilic compounds by mixed hemimicelles SPE technique is not a good strategy in the future, especially when surfactant and analytes are like charged. Also it is worth noting that although the two sub-types of compounds possess a large aromatic system, they still cannot obtain high recoveries by mixed hemimicelles SPE systems. These results suggested that π-π stacking interactions only plays a supporting role, and hydrophobic interaction may play the dominant role during the extraction process.

### Research on hydrophobic compounds excluding aromatic ring (retinyl acetate and myristic acid)

**Figure 9:**
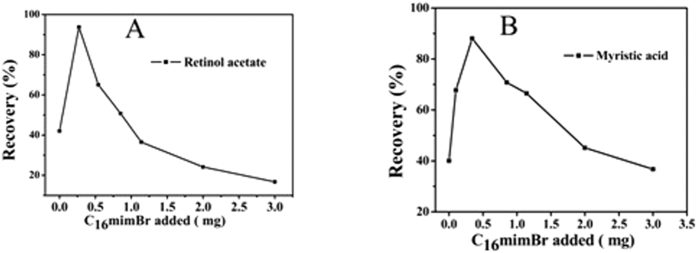
Effect of the amount of C_16_mimBr on the adsorption of Retinol acetate (**A**) and Myristic acid (**B**).

To further verify the dominant role of hydrophobic interaction and the supporting role of π-π stacking interactions, retinol acetate and myristic acid were investigated. It can be predicted that these two compounds hold hydrophobic interaction with the mixed hemimicelles because of the great hydrophobicity constant. The difference is that the retinol acetate contains long conjugate structure while myristic acid doesn’t. As shown in the Fig. 9, the retinol acetate and myristic acid were hardly adsorbed onto the surface of Fe_3_O_4_ NPs in the absence of the C_16_mimBr, then the adsorption ratio increased remarkably and finally decreased as the increasing of the amount of the C_16_mimBr, which is similar to the trend of the flavonoids and chlorophenol compounds. As shown in the Table 3, the two sub-types of the compounds have good extraction efficiency due to the high hydrophobic constant. The maximum recovery of retinol acetate is slightly higher than myristic acid, which implying that the conjugation reaction plays a little role in the extraction when the recoveries could reach a high level. These results verified the dominant role of hydrophobic interaction and the supporting role of conjugation reaction.

### Comparative research on two methods for flavonoids enrichment

In order to investigate the influence of hydrogen-bonding interaction, we studied two methods for flavonoids enrichment. Recently, some researchers have studied a two-step magnetic retrieval SPE technique in many matrices. Zhang *et al*. have applied the magnetic retrieval of chitosan solid phase extraction in green tea beverage samples^22^. Li *et al*. used the magnetic retrieval of ionic liquid in environmental water samples^23^. Therefore, we chose magnetic retrieval of chitosan solid-phase extraction technique and magnetic mixed hemimicelles solid-phase extraction technique for comparative research for flavonoids enrichment. It was found that the recoveries of flavonoids had obvious difference between the two methods. By using magnetic retrieval of chitosan solid-phase extraction, quercetin, luteolin and kaempferol had high extraction recoveries^24^. However, the recoveries of apigenin and chrysin were relatively low, which could only reach about 70% even under optimal extraction conditions. The main reason was that magnetic retrieval of chitosan solid-phase extraction process is mainly based on hydrogen-bonding interaction between chitosan and compounds. However, compared with other compounds, apigenin and chrysin have relatively fewer functional groups which can form hydrogen bonds. Therefore, the chitosan adsorption degrees of apigenin and chrysin were relatively low, resulting in relatively low extraction recoveries. However, by using magnetic mixed hemimicelles solid-phase extraction system, the phenomenon did not take place. The extraction recoveries of five flavonoids were very similar (Fig. 7), they could all achieve a relatively high level. Hence, the extraction mechanisms of magnetic retrieval of chitosan solid-phase extraction technique and magnetic mixed hemimicelles solid-phase extraction technique are different and hydrogen bonding interactions had no significant effect in the latter technique. This could be easily understood because magnetic mixed hemimicelles solid-phase extraction system did not contain a lot of hydrogen bonding sites. Based on this point, during the design process of magnetic mixed hemimicelles solid-phase extraction project, there is no need to consider hydrogen-bonding interaction, except the ionic liquid or ionic surfactants with a large amount of hydrogen bonding site.

### Comparative research on the structure of ionic liquids

#### Effect of different carbon chain lengths

**Figure 10:**
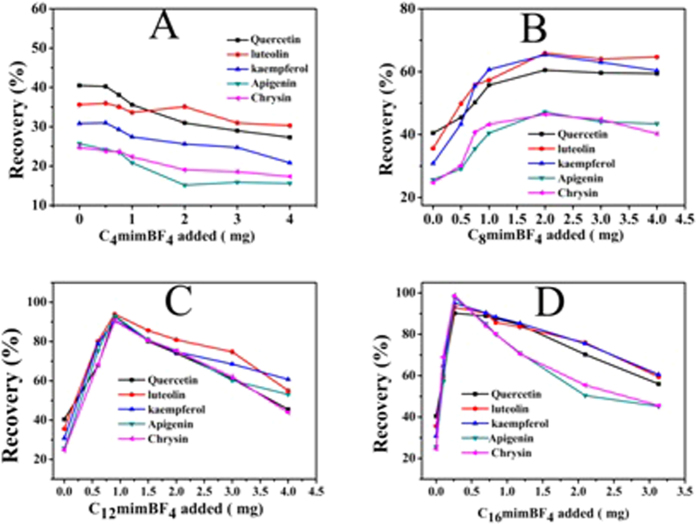
Effect of different lengths of carbon chain ionic liquid on the adsorption of five flavonoids: (**A**) C_4_mimBF_4_, (**B**) C_8_mimBF_4_, (**C**) C_12_mimBF_4_ and (**D**) C_16_mimBF_4_.

In this part, effect of different carbon chain lengths on the enrichment of five flavonoids was investigated based on the same parent nucleus structure and anionic species. Different chain lengths of ionic liquids (C_4_mimBF_4_, C_8_mimBF_4_, C_12_mimBF_4_ and C_16_mimBF_4_) on the surface of the Fe_3_O_4_ NPs were compared. The same as above, in the mixed hemimicelles region (below CMC) the sorbent offer three types of interactions: hydrophobic interactions from the hydrocarbon chain, π-π stacking interactions from aromatic ring and electrostatic attraction between C_16_minBr and flavonoids. As shown in Fig. 10(A), with the increasing amount of C_4_mimBF_4_, the recovery of five flavonoids became lower and lower, indicating that the C_4_mimBF_4_ is prone to interact with flavonoids in the aqueous solution, rather than being adsorbed onto the surface of the Fe_3_O_4_ NPs. With the increase of carbon chain length, the maximum recoveries of five flavonoids gradually increased and the required amount of ionic liquid to reach the maximum recovery gradually decreased. Therefore, the ionic liquids with a longer carbon chain are more suitable for magnetic mixed hemimicelles solid phase extraction. Because the longer carbon chain of ionic liquids are, the stronger hydrophobic interaction is. These results further confirm that the hydrophobic interactions play a dominant role in the magnetic mixed hemimicelles solid phase extraction system.

#### Effect of different parent nucleus structure

**Figure 11:**
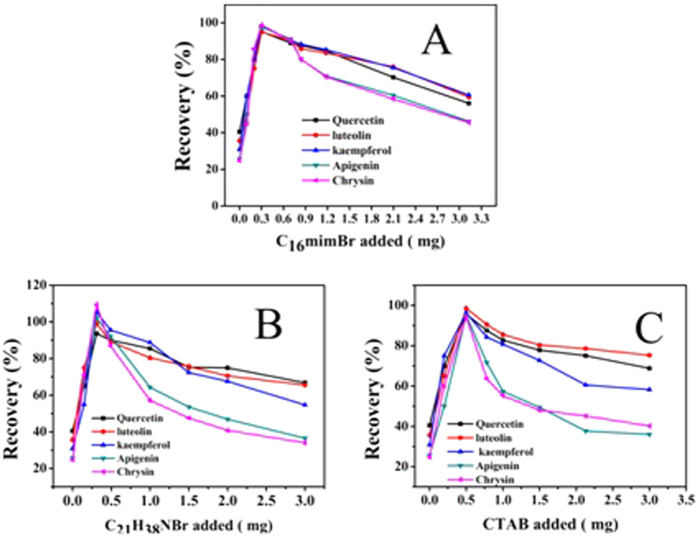
Effect of amount of ionic liquid and surfactants on the adsorption of five flavonoids compounds (**A**): C_16_mimBr (**B**) C_21_H_38_NBr and (**C**) CTAB.

In order to investigate the impact of the different parent nucleus structure of ionic liquids on the enrichment of flavonoids, two kinds of ionic liquids (C_16_mimBr, C_21_H_38_NBr) and surfactants cetyltrimethyl ammonium bromide (CTAB) were selected. As shown in Table 4, they have the same carbon chain length and the same anion, the only difference is their parent nucleus structure. Therefore, in the mixed hemimicelles region (below CMC) the π-π stacking interactions happened between ionic liquids and flavonoids, while CTAB didn’t. As can be seen from Fig. 11(A,B), only 0.3 mg C_16_mimBr and C_21_H_38_NBr and 0.5 mg surfactant CTAB were needed to achieve high recoveries, respectively. Very interestingly, they need relatively more amount of surfactant CTAB. After calculating and considering their relative molecular weight, we found it is reasonable to believe that ionic liquids containing aromatic action are more applicable to magnetic mixed hemimicelles solid phase extraction. This is important because it confirms once again from another angle that the π-π stacking interactions plays a supporting role in the magnetic mixed hemimicelles solid phase extraction system.

#### Effect of different anions

**Figure 12:**
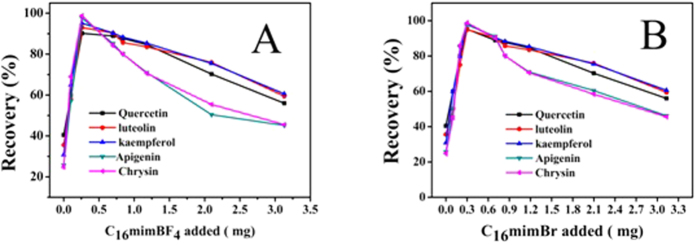
Effect of amount of ionic liquid on the adsorption of five flavonoids compounds (**A**): C_16_mimBF_4_ (**B**): C_16_mimBr.

In order to investigate the effect of different anions, we chose and compared two kinds of ionic liquids (C_16_mimBF_4_ and C_16_mimBr) with different anions. As shown in the Fig. 12(A) and (B), the five flavonoids can achieve high extraction efficiency. It can be drawn that different anions of the ionic liquid have no significant effect to form mixed hemimicelles solid phase extraction system.

After comparing the different types of ionic liquids with different carbon chain length, parent nucleus structure and different anions, it can be concluded that the carbon chain length of the ionic liquid plays a decisive role in forming mixed hemimicelles solid phase extraction system. The longer the carbon chain is, the easier to form the mixed hemimicelles solid phase extraction system. In another word, for the same analytes, the extraction efficiency increased obviously with the increase of hydrophobic constant of ionic liquid. We also found that the ionic liquid containing aromatic action is more suitable for magnetic mixed hemimicelles solid phase extraction system, which provides a guiding role in the process of choosing the ionic liquids.

### Optimization of extraction conditions

#### Effect of solution pH

Metallic oxide surface charge is an important factor in the formation of magnetic mixed hemimicelles solid-phase extraction adsorbents and extraction process. The pH value of the buffer plays a decisive role in the type and density of surface charge. While it will also make effects on the existing forms of analytes in the solution, thereby affecting the interaction between analytes and magnetic mixed hemimicelles solid-phase extracting agent.

**Figure 13:**
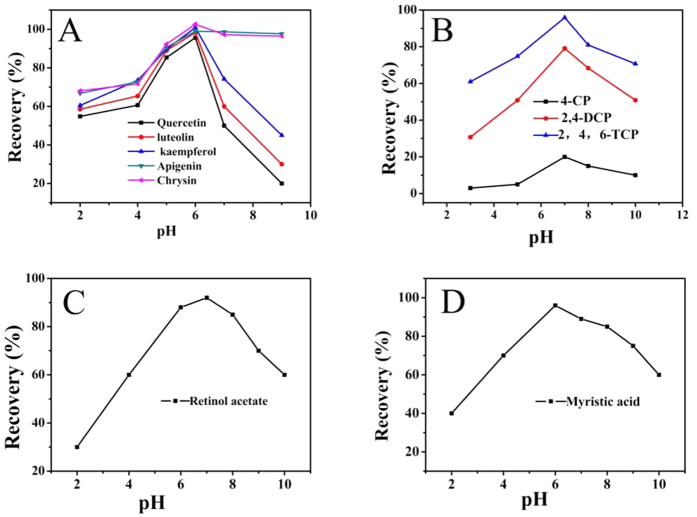
Effect of pH on the adsorption of flavonoids compounds (**A**), chlorophenol compounds (**B**), retinol acetate (**C**) and myristic acid (**D**).

As shown in Fig. 13(A), the effect of pH on enrichment of flavonoids and chlorophenol compounds was examined by varying the pH from 2.0 to 10.0. When the pH was around the IEP of Fe_3_O_4_ NPs, the charge density of Fe_3_O_4_ NPs surface was very low, and a small number of positive ions of C_16_mimBr were adsorbed onto the surface of Fe_3_O_4_ NPs to form mixed hemimicelles. When the pH value was above the IEP, the surfaces of Fe_3_O_4_ NPs became negatively charged, which led to strong electrostatic attraction between C_16_mimBr and the charged Fe_3_O_4_ NPs surface. The results indicated that the maximum adsorption performance occurred at pH 6.0, but when the pH was over 6.0, the recoveries of quercetin, luteolin and kaempferol decreased, while the recoveries of apigenin and chrysin remained unchanged. This can be explained by the fact that apigenin and chrysin posses higher hydrophobic constant (log P) and stronger hydrophobic interaction. On the other hand, because apigenin and chrysin have relatively high pKa, they still remain in molecular form when pH is high, therefore, the hydrophobic interaction with mixed micelles system is stronger, leading to higher extraction recoveries. As shown in Fig. 13(B), the recoveries of three chlorophenol compounds reached the maximum at pH 7.0. When the pH is above 7.0, the recoveries decreasd, which can be explained by the fact that the three compounds would be dissociated at higher pH conditions. Similarly, as shown in Fig. 13(C and D), the maximum adsorption of myristic acid and retinol acetate occurred at pH 6.0 and 7.0, respectively. From these results, It can be concluded that the recovery reduced when the analyte is dissociated. This is essential for researchers to quickly develop mixed hemimicelles solid phase extraction method. This result confirms once again from another perspective that hydrophobic interaction plays the dominant role in magnetic mixed hemimicelles solid phase extraction system.

#### Effect of kinds of magnetic supporting materials

Compared to the micro-sized solid-phase extraction support materials, magnetic nanoparticles have a high surface area and strong magnetism, therefore they are more suitable to construct magnetic solid phase extraction platform^25^,^26^. The magnetic nanomaterials (such as iron oxide magnetic nanoparticles) and magnetic composite materials (such as magnetic carbon nanotubes, magnetic graphene oxide) have been systematically compared in our group. The results showed that the advantage of magnetic composites is the greater surface area, and therefore the amount of adsorbents is less^12^. But the disadvantage is the complex synthetic process and high manufacturing cost. In this experiment, in order to explore the mechanism of magnetic mixed hemimicelles, we do not hope the support materials themselves have adsorption site of hydrophobic interaction, π-π stacking interactions, electrostatic interactions and hydrogen bonding interactions with analytes. So we chose iron oxide magnetic nanoparticles as a support material which has simple preparation process and a single type of surface functional groups, establishing magnetic mixed hemimicelles solid-phase extraction platform for the study of extraction mechanism.

#### Effect of the amount of Fe_3_O_4_ NPs

**Figure 14:**
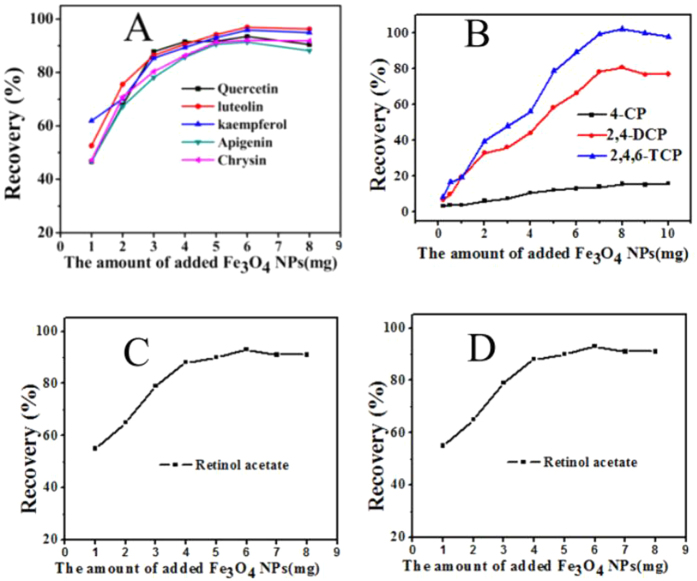
Effect of the amount of Fe_3_O_4_ NPs on the adsorption of (**A**) flavonoids compounds, (**B**) chlorophenol compounds, (**C**) Retinol acetateand (**D**) Myristic acid.

The designers should take into account that hemimicelles/mixed hemimicelles/admicelles are dynamic sorbents and the actual structure depends on the experimental conditions. If designers change the amount of nanoparticles while the amount of ionic liquid keeps constant, then the coverage of the surface and so the type of sorbent changed. Therefore, in order to optimize the amount of Fe_3_O_4_ NPs, we increased both Fe_3_O_4_ NPs and ionic liquid in the same proportion (20:1, mass ratio) in order to keep the type of sorbent. As shown in Fig. 14, through optimization, 6.0 mg Fe_3_O_4_ NPs and 0.3 mg C_16_mimBr were enough for total adsorption of flavonoids, myristic acid and retinol acetate, and 8.0 mg Fe_3_O_4_ NPs and 0.4 mg C_16_mimBr were enough for total adsorption of chlorophenol compounds of 100.0 ng mL^−1^ in 4 mL of sample solution. Further increasing the amounts of Fe_3_O_4_ NPs showed no significant improvement for the recoveries of analytes. It is worth noting that the amount of Fe_3_O_4_ NPs is quite small (If magnetic nanotubes are used as support materials, the amount is even smaller than 4 mg ref. 12). Considering that the current magnetic nanoparticles preparation methods generally have low yields^27^,^28^,^29^,^30^, there is few large-scale industrial productions, resulting in high purchase costs. However, in most cases, this method needs only 6.0 mg Fe_3_O_4_ NPs and 0.3 mg C_16_mimBr for 4 mL of sample solution and the total cost were less than 0.1 dollar. Therefore, compared with the conventional methods, less requirement on support material and surfactant is a significant advantage of magnetic mixed hemimicelles solid-phase extraction technique^31^,^32^,^33^.

#### Effect of ionic strength

The ionic strength influence the surface charge density. Therefore, we studied the effect of ionic strength by varying the sodium ions concentrations between 0.02 and 0.5 mol/L. With the increase of the ionic strength, recoveries of the analytes decreased. Therefore, 0.02 mol/L was selected for the further studies.

#### Effect of extraction time

In order to obtain satisfactory recoveries, the effect of extraction time on adsorption and magnetic separating time were investigated under the optimal conditions mentioned above. The effect of extraction time on the adsorption was examined by changing the time from 5 to 120 min. The results suggested that 10 min was sufficient for the highest extraction recoveries. However, when the extraction time was more than 10 min, recoveries of analytes decreased. This phenomenon may be attributed to the fact that with the increase of extraction time, some ionic liquid or ionic surfactants redissolved into the solution. Therefore, 10 min was selected for extraction time, which enabled these analytes to be completely adsorbed.

#### Effect of desorption conditions

To obtain satisfactories recovery of the above there categories of analytes, different kinds of organic solvents (methanol, methanol containing 1% HAc, methanol containing 2% HAc, acetonitrile, acetonitrile containing 1% HAc and acetonitrile containing 2% HAc) which were widely used to completely disrupt the hemimicelles and admicelles were investigated. We found that methanol containing 1% HAc had the best desorption ability. This may be attributed to the fact that the addition of acetic acid caused the analytes to exist in the molecular form which was prone to be soluble in organic solvent. Besides, when the pH value of the desorption solvent was below or around the IEP of Fe_3_O_4_ NPs, charge density on Fe_3_O_4_ NPs surface was low, which was favorable for the disruption of mixed hemimicelles.

#### Method validation

According to the above experiments, we found that the four sub-types of hydrophobic compounds including flavonoids, chlorophenol compounds, retinol acetate and myristic acid can achieve good extraction efficiency in the magnetic mixed hemimicelles solid phase extraction system. Therefore, to evaluate the accuracy and feasibility of the magnetic mixed hemimicelles SPE platform developed, the four sub-types of hydrophobic compounds were enriched and detected in complex samples.

**Figure 15:**
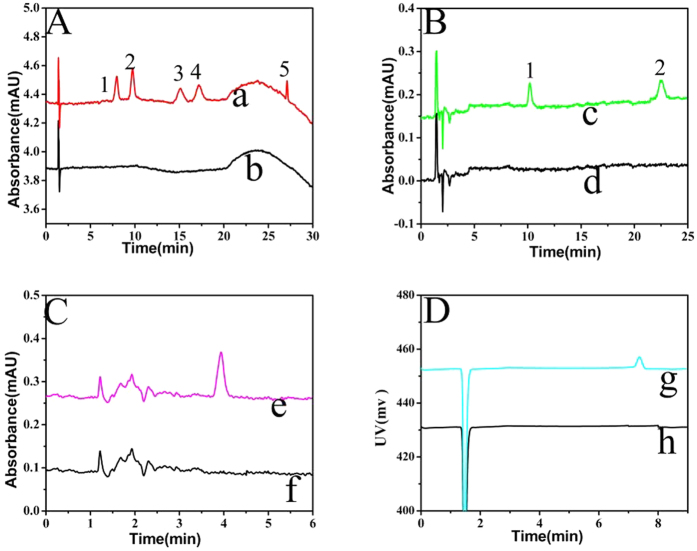
HPLC chromatogram of compounds which were spiked in aqueous system samples and extracted by SPE at optimum conditions, (**A**): (a) concentration of each analyte: 20.0 ng mL^−1^; (b) blank urine sample (Compound number: 1. quercetin; 2. luteolin; 3. Kaempferol; 4. Apigenin; 5. chrysin), (**B**): (c) concentration of each analyte: 100.0 ng mL^−1^; (d) blank tap water sample (Compound number: 1. 2, 4-DCP; 2. 2, 4, 6-TCP); (**C**): (e) concentration of Retinol acetate: 20.0 ng mL^−1^; (f) blank urine sample; (**D**): (g) concentration of Myristic acid: 1 mg ml^−1^; (h) blank urine sample.

Firstly, the magnetic mixed hemimicelles SPE was applied in the enrichment of flavonoids in the biological system. 6.0 mg Fe_3_O_4_ NPs and 0.3 mg C_16_mimBr were added into urine samples, extraction time was 10 min, and methanol containing 1% HAc was used for the desorption. To evaluate the accuracy and precision of the method, urine samples spiked with the five flavonoids were analyzed. The samples were dealt with the proposed MSPE method before injection. Chromatogram was shown in Fig. 15(A). It can be seen that no interfering peaks at the elution times of analytes in urine samples were observed. The retention times of quercetin, luteolin, kaempferol, apigenin and chrysin were 7.96, 9.35, 15.17, 17.23, 26.93 min respectively. The results implied the proposed method based on C_16_mimBr-coated Fe_3_O_4_ NPs was appropriate for the determination of quercetin, luteolin, kaempferol, apigenin and chrysin.

Secondly, the MSPE was applied in the enrichment of chlorophenol compounds in the environmental system. 8.0 mg Fe_3_O_4_ NPs and 0.4 mg C_16_mimBr were added into environmental samples, extraction time was 10 min, and methanol containing 1% HAc was used for the desorption. To evaluate the accuracy and precision, tap water samples spiked with the 2,4-dichlorophenol and 2,4,6-trichlorophenol were analyzed. The samples were dealt with the proposed MSPE method before injection. Chromatogram was shown in Fig. 15(B). The retention times of 2,4-dichlorophenol, 2,4,6-trichlorophenol were 10.19 and 22.56 min respectively. The results implied the proposed method based on C_16_mimBr-coated Fe_3_O_4_ NPs was appropriate for the determination of 2,4-dichlorophenol and 2,4, 6-trichlorophenol.

Finally, the MSPE was applied in the enrichment of retinol acetate and myristic compounds in biological systems. 6.0 mg Fe_3_O_4_ NPs and 0.3 mg C_16_mimBr were added into urine samples, extraction time was 10 min, and methanol containing 1% HAc was used for the desorption. To evaluate the accuracy and precision, urine samples spiked with the retinol acetate and myristic were analyzed. The samples were dealt with the proposed MSPE method before injection. Chromatogram was shown in Fig. 15(C,D). It can be seen that no interfering peaks at the elution times of analytes in urine samples were observed. The retention times of retinol acetate and myristic were 3.93 and 7.45 min respectively. The results implied the proposed method based on C_16_mimBr-coated Fe_3_O_4_ NPs was appropriate for the determination of retinol acetate and myristic in biological systems.

#### Linearity, LOD and LOQ

As is shown in Table 6, under the optimized conditions, the quantitative parameters of the proposed method, including linear range, correlation coefficients were evaluated. The correlation coefficient (R^2^) ranged from 0.9912 to 0.9992. The LOD and LOQ were considered as the analyte minimum concentrations that can be confidently identified and quantified by the method. The LOD calculated on the basis of signal-to-noise ratio of 3 (S/N = 3) for luteolin can be achieved to 0.5 ng mL^−1^.

#### Precision

The precision were evaluated by applying the proposed magnetic mixed hemimicelles SPE method to six replicate spiked samples at three different concentration levels (low, middle and high quantification concentrations). The precision results were shown in Table 7. These results demonstrated that the magnetic mixed hemimicelles SPE method had good precision.

#### Recovery

The recovery of the method was assessed by using the standard addition method. Table 8 showed the recoveries that were calculated after spiking three different concentration levels (low, middle and high quantification concentrations), respectively. The recoveries were expressed as the mean value of three independent determinations. Recoveries were in the range of 80.1–103.7%, with the relative standard deviations of the recoveries varying between 2.0% and 8.7%. These results demonstrated the method was suitable for analyzing the four sub-types of hydrophobic compounds in complex samples.

#### The extraction mechanism of magnetic mixed hemimicelles SPE system

This work developed a series of simple, cheap and accurate extraction methods for the determination of trace amounts of compounds in complex samples. Most importantly, we hope to, through our systematic study, firstly illustrate the extraction mechanism of magnetic mixed hemimicelles SPE system. The results were summed up as follows:

In order to study the influence of hydrophobic interaction, four sub-types of hydrophobic compounds including flavonoids, chlorophenols, retinol acetate and myristic acid were selected and examined. The results demonstrated that the hydrophobic interaction plays a dominate role in the magnetic mixed hemimicelles solid phase extraction. For different analytes, the extraction efficiency increased obviously with the increase of hydrophobic constant of analytes. For the same analytes, the extraction efficiency increased obviously with the increase of hydrophobic constant of ionic liquid (C_16_mimBr, C_12_mimCl, C_12_mimBF_4_) or ionic surfactants (such as sodium dodecyl sulfate or cetyltrimethylammonium bromide).

In order to study the effect of π-π stacking interactions, two sub-types of hydrophobic compounds consisting of flavonoids and chlorophenols were compared, the extraction efficiency increased with the larger conjugation structure, which indicated that π-π stacking interactions plays a role in the extraction process. However, it is worth noting that although quinolone and cephalosporin compounds possess a large aromatic system, they still cannot obtain high recoveries by mixed hemimicelles SPE systems. These results suggested that π-π stacking interactions only plays a supporting role.

In order to study the effect of hydrogen-bonding interaction, magnetic mixed hemimicelles SPE method and magnetic retrieval of chitosan SPE method were compared to extract five flavonoids compounds. It was found that the recoveries of flavonoids had clear difference in magnetic retrieval of chitosan solid-phase extraction. The compounds contain smaller groups which can make hydrogen bonds, and the extraction recoveries are lower. The main reason was that magnetic retrieval of chitosan solid-phase extraction process is mainly based on hydrogen-bonding interaction between chitosan and compounds. However, in magnetic mixed hemimicelles solid-phase extraction system, the extraction recoveries of five flavonoids had little difference, which indicated that hydrogen bonding interactions in magnetic mixed hemimicelles solid-phase extraction system had no significant effect, except the ionic liquid or ionic surfactants with a large amount of hydrogen bonding site.

In order to study the effect of electrostatic interaction, two sub-types of hydrophilic cephalosporins and quinolones compounds were chosen and investigated. Interestingly, compared with the pure Fe_3_O_4_ NPs, the recoveries of these hydrophilic compounds decreased observably when mixed hemimicelles were formed by adding ionic liquids, revealing a very useful rule that trying to analyze hydrophilic compounds by mixed hemimicelles SPE technique is not a good strategy in the future, especially when surfactant and analytes are like charged.

In a word, the extraction mechanism may be speculated that hydrophobic interaction plays a dominant role, while π-π stacking interactions plays a supporting role, hydrogen bonds usually do not play a role, electrostatic interaction sometimes even plays as negative antagonism (electrostatic repulsion) in the magnetic mixed hemimicelles solid phase extraction. Therefore, if researchers need to develop an analysis method based on magnetic mixed hemimicelles solid phase extraction technique, it is essential to grasp these simple guidelines: if log P < 0, hydrophilic compound is not suitable for the magnetic mixed hemimicelles solid phase extraction because of the absence of hydrophobic interaction; if 0 ≤ log P ≤ 3, weak hydrophobic compound may be suitable for the magnetic mixed hemimicelles solid phase extraction system under the π-π stacking interactions reaction; if Log P > 3, strong hydrophobic compound is suitable for the magnetic mixed hemimicelles solid phase extraction system on the basis of a strong hydrophobic interaction. It is worth noting that these values are empirical data which are speculated according to the experimental results. More accurate values are pending for efforts of more researchers and supports by more experimental data.

#### The application guidelines of magnetic mixed hemimicelles SPE platform

To construct mixed hemimicelles SPE platform, supporting materials, surfactants and sample matrix should be noticed except considering properties of analytes. Therefore, we summarized representative literature of mixed hemimicelles SPE in Table 9 and provided the following recommendations:

For support materials, compared with conventional support materials, some nanomaterials (such as carbon nanotubes and graphene oxide) have obvious advantages in saving the dosage based on their high specific surface area^12^. Moreover, when selecting some multifunctional support materials, especially magnetic materials for example, the magnetic mixed hemimicelles SPE platform can be easily collected and separated by an external magnetic field without additional centrifugation or filtration, which makes separation easier and faster.

For surfactants, this work revealed a rule that the extraction efficiency of analytes increased obviously with the increase of hydrophobic constant of ionic liquid (mainly depends on the length of the carbon chain). This rule might be applicable to other ionic liquid (C_16_mimBr, C_12_mimCl, C_12_mimBF_4_) or ionic surfactants (such as sodium dodecyl sulfate or cetyltrimethylammonium bromide). It’s also found the ionic liquid containing π-π stacking interactions is more suitable for magnetic mixed hemimicelles solid phase extraction system.

For sample matrix, the mixed hemimicelles SPE platform might be applicable to analyze samples in aqueous matrix (hydrophilic), rather than oily matrix (hydrophobic). Because based on our group experience and literature^12^,^18^ in this area (as summarized in Table 9), It’s found mixed hemimicelles SPE method have been successfully applied in aqueous matrix (such as water, sewage, urine, serum, plasma, red wine). Interestingly, so far there has no application in oily matrix (such as oil, plant oil, gasoline). Actually this is understandable that, compared with in aqueous matrix, the hydrophobic interaction between analytes and mixed hemimicelles are competition and interference in oily matrix.

## Conclusions

The formation of magnetic mixed hemimicelles is a valuable strategy for the extraction and concentration of analytes in complex samples. This work developed a series of simple, cheap and accurate extraction methods for the determination of three categories and six sub-types of compounds. Most importantly, to the best of our knowledge, this is the first attempt to systematically study and comprehensively illustrate the extraction mechanism of mixed hemimicelles solid-phase extraction. The application guidelines about supporting materials, surfactants and sample matrix were also summarized. With the extraction mechanism and platform established in the study, confidence is gained and increased purposefulness is achieved, providing a stepping stone in the realization of the widespread application of magnetic mixed hemimicelles solid-phase extraction for preconcentration of trace amounts of analytes from complex samples.

## Additional Information

**How to cite this article**: Xiao, D. *et al*. Platform construction and extraction mechanism study of magnetic mixed hemimicelles solid-phase extraction. *Sci. Rep.* **6**, 38106; doi: 10.1038/srep38106 (2016).

**Publisher's note:** Springer Nature remains neutral with regard to jurisdictional claims in published maps and institutional affiliations.

## Figures and Tables

**Table 1:**
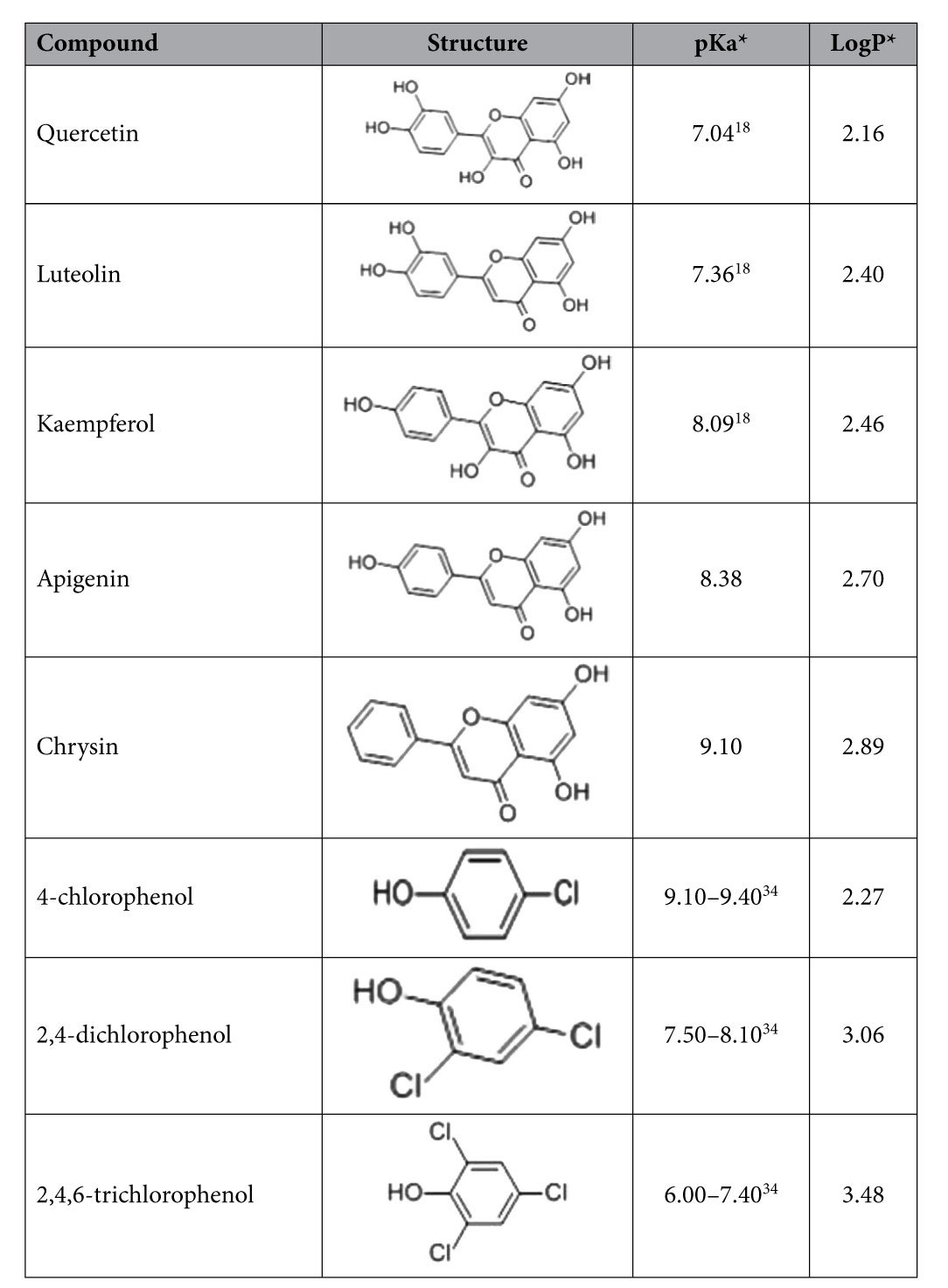
Basic information of hydrophobic drugs containing large aromatic system.

^*^The value of pKa (acid dissociation constant) and LogP (hydrophobic constant) in Table 1 to Table 4 are obtained by literatures or https://pubchem.ncbi.nlm.nih.gov/ or http://www.drugbank.ca/ or http://www.molbase.com/.

**Table 2:**
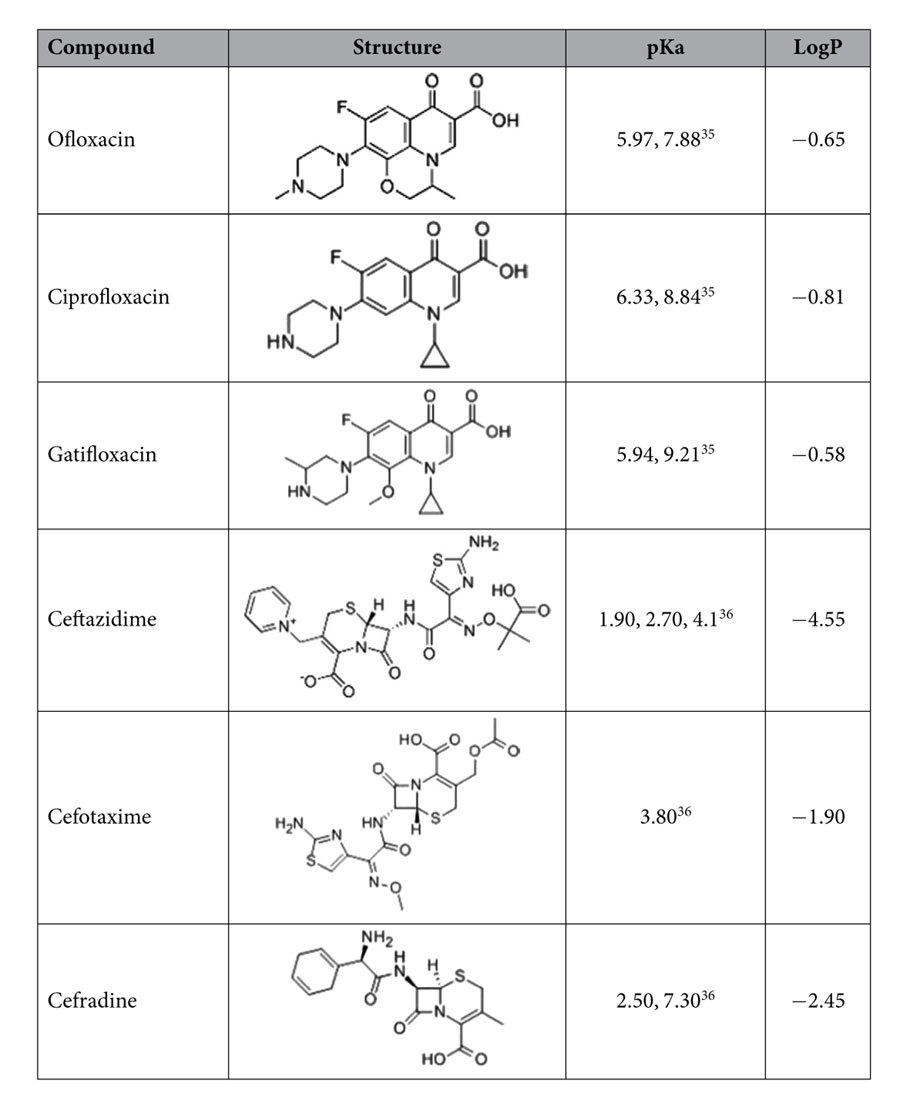
Basic information of hydrophilic drugs containing large aromatic system.

**Table 3:**
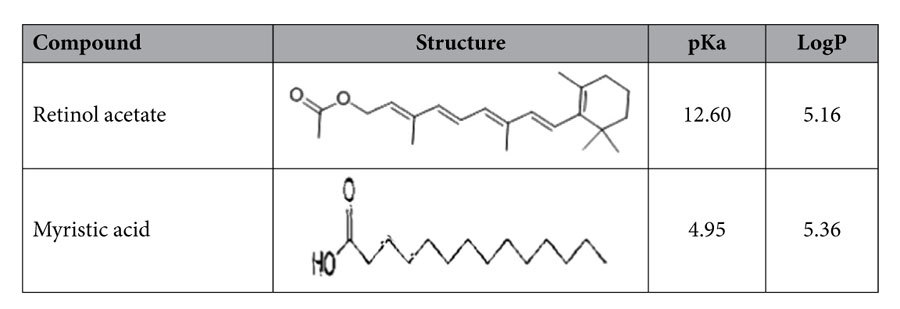
Basic information of hydrophobic drugs excluding large aromatic system.

**Table 4:**
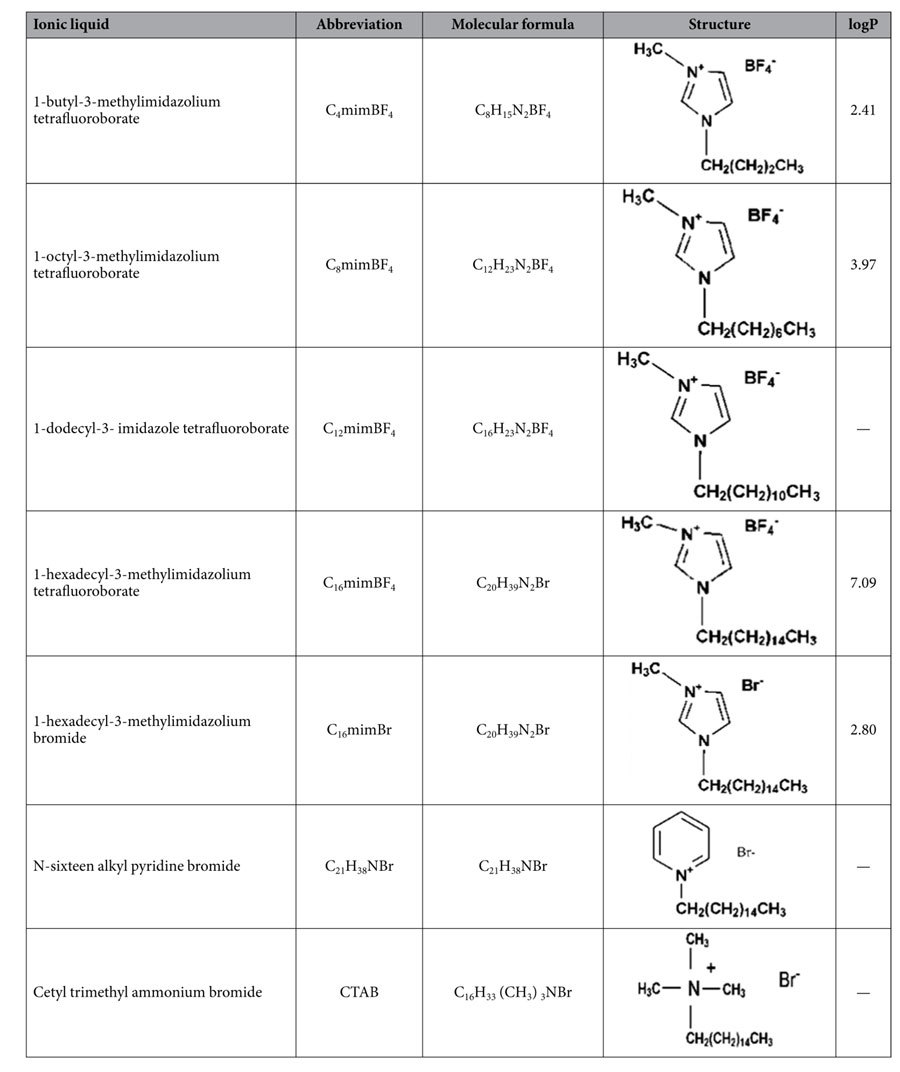
Chemical structure of RTILs and ionic surfactants.

**Table 5:** General chromatographic conditions.

Analytes	Mobile phase	Column temperature (°C)	Detection wavelength (nm)	Flow rate (ml min^−1^)	Injection volume (μL)
Flavonoids	Gradient elution	30	360	1.0	10
Chlorophenols	Methanol-0.1% formic acid	30	254	1.0	10
Fluoroquinolones	Methanol - 1% aqueous solution of triethylamine	30	325	1.0	10
Cephalosporins	Methanol-0.1% formic acid	30	254	1.0	10
Vitamin A acetate	methanol- water	30	325	1.0	10
Myristic acid	methanol- water	30	—	1.0	20

**Table 6:** Analytical parameters of the proposed method.

Analytes	Liner range (ng/ml)	R^2^	LOD (ng/ml)	LOQ (ng/ml)
Quercetin	2.0–1500	0.9912	0.7	2.0
Luteolin	1.5–1500	0.9987	0.5	1.5
Kaempferol	3.0–1500	0.9946	1.0	3.0
Apigenin	3.5–1500	0.9990	1.2	3.5
Chrysin	2.5–1500	0.9991	0.9	2.5
2,4-dichlorophenol	35–1500	0.9976	10	35
2,4,6-trichlorophenol	20–1500	0.9992	6	20
Retinyl acetate	2.0–1500	0.9985	0.5	2.0
Myristic acid	700–40000	0.9991	200	700

**Table 7:** Precision and accuracy for detection of five flavonoids, chlorophenol, retinol acetate and myristic acid in samples.

Analytes	Conc. (ng/mL)	Mean accuracy (%)	RSD (%)
Quercetin	20	95.0	5.2
	100	98.5	5.6
	500	101.0	6.3
Luteolin	20	98.0	2.8
	100	96.5	4.8
	500	95.6	3.6
Kaempferol	20	102.0	3.0
	100	101.8	5.7
	500	99.7	6.9
Apigenin	20	101.0	1.9
	100	99.4	3.1
	500	96.3	5.4
Chrysin	20	99.5	4.9
	100	100.5	6.4
	500	99.1	8.5
2,4-dichlorophenol	50	80.4	8.0
	200	85.6	4.3
	1000	79.8	2.9
2,4,6-trichlorophenol	20	99.0	2.0
	100	97.9	4.3
	1000	100.7	2.9
Retinyl acetate	20	90.7	4.3
	100	96.4	3.2
	1000	91.5	2.1
Myristic acid	1000	88.9	3.6
	5000	86.7	3.4
	20000	87.2	7.0

**Table 8:** Recovery for five flavonoids, chlorophenol, retinol acetate and myristic acid in samples.

Analytes	Conc. (ng/mL)	Mean accuracy (%)	RSD (%)
Quercetin	20	94.0	3.2
	100	96.7	4.6
	500	103.7	5.3
Luteolin	20	96.0	2.8
	100	98.5	4.0
	500	100.7	4.6
Kaempferol	20	101.0	7.0
	100	92.9	6.9
	500	98.6	4.9
Apigenin	20	98.7	2.0
	100	102.8	2.9
	500	96.7	3.4
Chrysin	20	99.7	3.7
	100	97.9	6.7
	500	99.6	8.7
2,4-dichlorophenol	50	80.1	2.9
	200	82.6	5.0
	1000	80.6	3.8
2,4,6-trichlorophenol	20	101.0	4.0
	100	98.6	5.3
	1000	100.4	3.9
Retinyl acetate	20	90.4	4.3
	100	93.4	3.2
	1000	92.5	2.1
Myristic acid	1000	88.0	2.6
	5000	87.7	5.4
	20000	86.2	6.0

**Table 9:** Comparison of different method applied magnetic mixed hemimicelles SPE technique.

Sample matrix	Surpport materials	Surfactants	Representative analytes	log P	pKa	Extraction pH	LOD (ng mL^−1^)	Recovery (%)	RSD (%)	Ref.
Urine	MCNTs	C_16_mimBr	Luteolin	2.40	7.36	7.0	0.20	98.3–107.5	3.5–4.0	12
Beer	Fe_3_O_4_/SiO_2_ NPs	CTAB	xanthohumol	4.22	—	7.0	0. 6	93–103	4.4–7.3	37
Environmental water	Fe_3_O_4_ NPs	C_16_mimBr	2,4-dichlorophenol	3.06	7.89	10.0	0.12	74–90	6.2	38
Water and soil	Fe_3_O_4_ NPs	C16mimBr	Pyrene	4.88	—	10.0	8330	76–96	4.6	13
Environmental water	Fe_3_O_4_/Al_2_O_3_ NPs	SDS	Trimethoprim	0.91	7.12	2.0	0.09	67–86	2–6	39
Environmental water	Fe_3_O_4_ NPs	SDS	levofloxacin	−0.39	6.24	7.0	0.1	79–114*	3.5–3.8	40
Urine	Fe_3_O_4_ NPs	C_16_mimBr	Quercetin	2.16	7.04	6.0	0.7	94.0–103.7	3.2–5.3	This method

^*^Absolute recovery was only 20–40%, implying hydrophilic compound is not suitable for the magnetic mixed hemimicelles SPE technique.

## References

[b1] Luque N., Merino F., Rubio S. & Perez-Bendito D. Stability of benzalkonium surfactants on hemimicelle-based solid-phase extraction cartridges. Journal of Chromatography A 1094, 17–23, doi: 10.1016/j.chroma.2005.07.102 (2005). 16257284

[b2] Moral A., Sicilia M. D., Rubio S. & Perez-Bendito D. Sodium dodecyl sulphate-coated alumina for the extraction/preconcentration of benzimidazolic fungicides from natural waters prior to their quantification by liquid chromatography/fluorimetry. Analytica chimica acta 569, 132–138, doi: 10.1016/j.aca.2006.03.069 (2006).

[b3] Niu H. . Cetyltrimethylammonium bromide-coated titanate nanotubes for solid-phase extraction of phthalate esters from natural waters prior to high-performance liquid chromatography analysis. Journal of Chromatography A 1172, 113–120, doi: 10.1016/j.chroma.2007.10.014 (2007). 17963775

[b4] Saitoh T., Yamaguchi M. & Hiraide M. Surfactant-coated aluminum hydroxide for the rapid removal and biodegradation of hydrophobic organic pollutants in water. Water Research 45, 1879–1889, doi: 10.1016/j.watres.2010.12.009 (2011). 21193213

[b5] Amjadi M. & Samadi A. Modified ionic liquid-coated nanometer TiO2 as a new solid phase extraction sorbent for preconcentration of trace nickel. Colloids and Surfaces a-Physicochemical and Engineering Aspects 434, 171–177, doi: 10.1016/j.colsurfa.2013.04.059 (2013).

[b6] Merino F., Rubio S. & Perez-Bendito D. Solid-phase extraction of amphiphiles based on mixed hemimicelle/admicelle formation: Application to the concentration of benzalkonium surfactants in sewage and river water. Analytical Chemistry 75, 6799–6806, doi: 10.1021/ac030224a (2003). 14670038

[b7] Cantero M., Rubio S. & Perez-Bendito D. Determination of alkylphenols and alkylphenol carboxylates in wastewater and river samples by hemimicelle-based extraction and liquid chromatography-ion trap mass spectrometry. Journal of Chromatography A 1120, 260–267, doi: 10.1016/j.chroma.2005.12.048 (2006). 16412449

[b8] Li J., Cai Y., Shi Y., Mou S. & Jiang G. Analysis of phthalates via HPLC-UV in environmental water samples after concentration by solid-phase extraction using ionic liquid mixed hemimicelles. Talanta 74, 498–504, doi: 10.1016/j.talanta.2007.06.008 (2008). 18371667

[b9] Ma N. . Magnetic solid-phase extraction based on a trimethylstearylammonium bromide coated Fe3O4/SiO2 composite for determination of adriamycin hydrochloride in human plasma and urine by HPLC-FLD. Analytical Methods 6, 6736–6744, doi: 10.1039/c4ay00768a (2014).

[b10] Ding J., Zhao Q., Sun L., Ding L. & Ren N. Magnetic mixed hemimicelles solid-phase extraction of xanthohumol in beer coupled with high-performance liquid chromatography determination. Journal of Separation Science 34, 1463–1468, doi: 10.1002/jssc.201000930 (2011). 21548085

[b11] Liu Q. . Hemimicelles/admicelles supported on magnetic graphene sheets for enhanced magnetic solid-phase extraction. Journal of Chromatography A 1257, 1–8, doi: 10.1016/j.chroma.2012.08.028 (2012). 22921358

[b12] Xiao D. . Mixed hemimicelle solid-phase extraction based on magnetic carbon nanotubes and ionic liquids for the determination of flavonoids. Carbon 72, 274–286, doi: 10.1016/j.carbon.2014.01.075 (2014).

[b13] Zhang Q. . Ionic liquid-coated Fe3O4 magnetic nanoparticles as an adsorbent of mixed hemimicelles solid-phase extraction for preconcentration of polycyclic aromatic hydrocarbons in environmental samples. Analyst 135, 2426–2433, doi: 10.1039/c0an00245c (2010). 20668754

[b14] Li J. . Mixed hemimicelles solid-phase extraction based on cetyltrimethylammonium bromide-coated nano-magnets Fe3O4 for the determination of chlorophenols in environmental water samples coupled with liquid chromatography/spectrophotometry detection. Journal of Chromatography A 1180, 24–31, doi: 10.1016/j.chroma.2007.12.028 (2008). 18179801

[b15] Sun L. . Analysis of sulfonamides in environmental water samples based on magnetic mixed hemimicelles solid-phase extraction coupled with HPLC-UV detection. Chemosphere 77, 1306–1312, doi: 10.1016/j.chemosphere.2009.09.049 (2009). 19836824

[b16] Zhu L. . Mixed hemimicelles SPE based on CTAB-coated Fe3O4/SiO2 NPs for the determination of herbal bioactive constituents from biological samples. Talanta 80, 1873–1880, doi: 10.1016/j.talanta.2009.10.037 (2010). 20152426

[b17] Cheng Q., Qu F., Li N. B. & Luo H. Q. Mixed hemimicelles solid-phase extraction of chlorophenols in environmental water samples with 1-hexadecyl-3-methylimidazolium bromide-coated Fe3O4 magnetic nanoparticles with high-performance liquid chromatographic analysis. Analytica chimica acta 715, 113–119, doi: 10.1016/j.aca.2011.12.004 (2012). 22244175

[b18] He H. . Mixed hemimicelles solid-phase extraction based on ionic liquid-coated Fe3O4/SiO2 nanoparticles for the determination of flavonoids in bio-matrix samples coupled with high performance liquid chromatography. Journal of Chromatography A 1324, 78–85, doi: 10.1016/j.chroma.2013.11.021 (2014). 24290172

[b19] Xiao D. . Magnetic carbon nanotubes: synthesis by a simple solvothermal process and application in magnetic targeted drug delivery system. Journal of Nanoparticle Research 14, doi: 10.1007/s11051-012-0984-4 (2012).

[b20] Zhao X., Shi Y., Ca Y. & Mou S. Cetyltrimethylammonium bromide-coated magnetic nanoparticles for the preconcentration of phenolic compounds from environmental water samples. Environmental Science & Technology 42, 1201–1206, doi: 10.1021/es071817w (2008). 18351093

[b21] Zhao X., Shi Y., Wang T., Cai Y. & Jiang G. Preparation of silica-magnetite nanoparticle mixed hemimicelle sorbents for extraction of several typical phenolic compounds from environmental water samples. Journal of Chromatography A 1188, 140–147, doi: 10.1016/j.chroma.2008.02.069 (2008). 18329033

[b22] Zhang H.-F. & Shi Y.-P. Magnetic retrieval of chitosan: Extraction of bioactive constituents from green tea beverage samples. Analyst 137, 910–916, doi: 10.1039/c1an15873b (2012). 22167525

[b23] Zhang J. . Magnetic retrieval of ionic liquids: Fast dispersive liquid-liquid microextraction for the determination of benzoylurea insecticides in environmental water samples. Journal of Chromatography A 1254, 23–29, doi: 10.1016/j.chroma.2012.07.051 (2012). 22871379

[b24] Xiao D. . Magnetic solid-phase extraction based on Fe3O4 nanoparticle retrieval of chitosan for the determination of flavonoids in biological samples coupled with high performance liquid chromatography. Rsc Advances 4, 64843–64854, doi: 10.1039/c4ra13369b (2014).

[b25] Zhang J. . Application of ionic-liquid-supported magnetic dispersive solid-phase microextraction for the determination of acaricides in fruit juice samples. Journal of Separation Science 36, 3249–3255, doi: 10.1002/jssc.201300358 (2013). 23894018

[b26] Rajabi A. A., Yamini Y., Faraji M. & Seidi S. Solid-phase microextraction based on cetyltrimethylammonium bromide-coated magnetic nanoparticles for determination of antidepressants from biological fluids. Medicinal Chemistry Research 22, 1570–1577, doi: 10.1007/s00044-012-0158-z (2013).

[b27] Ramimoghadam D., Bagheri S. & Hamid S. B. A. Progress in electrochemical synthesis of magnetic iron oxide nanoparticles. Journal of Magnetism and Magnetic Materials 368, 207–229, doi: 10.1016/j.jmmm.2014.05.015 (2014).

[b28] Qu J.-B., Shao H.-H., Jing G.-L. & Huang F. PEG-chitosan-coated iron oxide nanoparticles with high saturated magnetization as carriers of 10-hydroxycamptothecin: Preparation, characterization and cytotoxicity studies. Colloids and Surfaces B-Biointerfaces 102, 37–44, doi: 10.1016/j.colsurfb.2012.08.004 (2013). 23000675

[b29] Liu T., Chang G., Cao R. & Meng L. Applications of Superparamagnetic Fe3O4 Nanoparticles in Magnetic Resonance Imaging. Progress in Chemistry 27, 601–613, doi: 10.7536/pc141042 (2015).

[b30] Yao H., Fan M., Wang Y., Luo G. & Fei W. Magnetic titanium dioxide based nanomaterials: synthesis, characteristics, and photocatalytic application in pollutant degradation. Journal of Materials Chemistry A 3, 17511–17524, doi: 10.1039/c5ta03215f (2015).

[b31] Sun Y. . One pot synthesis of magnetic graphene/carbon nanotube composites as magnetic dispersive solid-phase extraction adsorbent for rapid determination of oxytetracycline in sewage water. Journal of chromatography. A 1422, 53–59, doi: 10.1016/j.chroma.2015.10.035 (2015). 26518491

[b32] Zhao J., Liao W. & Yang Y. Magnetic solid-phase extraction for determination of sulpiride in human urine and blood using high-performance liquid chromatography. Biomedical Chromatography 29, 1871–1877, doi: 10.1002/bmc.3509 (2015). 26019021

[b33] Trujillo-Rodriguez M. J., Pino V., Anderson J. L., Ayala J. H. & Afonso A. M. Double salts of ionic-liquid-based surfactants in microextraction: application of their mixed hemimicelles as novel sorbents in magnetic-assisted micro-dispersive solid-phase extraction for the determination of phenols. Analytical and bioanalytical chemistry 407, 8753–8764, doi: 10.1007/s00216-015-9034-2 (2015). 26403236

[b34] Czaplicka M. Sources and transformations of chlorophenols in the natural environment. Science of the Total Environment 322, 21–39, doi: 10.1016/j.scitotenv.2003.09.015 (2004). 15081735

[b35] He K. & Blaney L. Systematic optimization of an SPE with HPLC-FLD method for fluoroquinolone detection in wastewater. Journal of Hazardous Materials 282, 96–105, doi: 10.1016/j.jhazmat.2014.08.027 (2015). 25200119

[b36] Wang P., Yuan T., Hu J. & Tan Y. Determination of cephalosporin antibiotics in water samples by optimised solid phase extraction and high performance liquid chromatography with ultraviolet detector. International Journal of Environmental Analytical Chemistry 91, 1267–1281, doi: 10.1080/03067311003778649 (2011).

[b37] Ding J., Zhao Q., Sun L., Ding L. & Ren N. Magnetic mixed hemimicelles solid-phase extraction of xanthohumol in beer coupled with high-performance liquid chromatography determination. J Sep Sci 34, 1463–1468, doi: 10.1002/jssc.201000930 (2011). 21548085

[b38] Cheng Q., Qu F., Li N. B. & Luo H. Q. Mixed hemimicelles solid-phase extraction of chlorophenols in environmental water samples with 1-hexadecyl-3-methylimidazolium bromide-coated Fe3O4 magnetic nanoparticles with high-performance liquid chromatographic analysis. Anal Chim Acta 715, 113–119, doi: 10.1016/j.aca.2011.12.004 (2012). 22244175

[b39] Sun L. . Preparation of alumina-coated magnetite nanoparticle for extraction of trimethoprim from environmental water samples based on mixed hemimicelles solid-phase extraction. Anal Chim Acta 638, 162–168, doi: 10.1016/j.aca.2009.02.039 (2009). 19327455

[b40] Wang L., Yuan Q., Liang G., Shi L. & Zhan Q. Magnetic mixed hemimicelles solid-phase extraction coupled with high-performance liquid chromatography for the extraction and rapid determination of six fluoroquinolones in environmental water samples. J Sep Sci 38, 996–1001, doi: 10.1002/jssc.201401216 (2015). 25581496

